# Musical preferences of Brazilian high school students

**DOI:** 10.1371/journal.pone.0239891

**Published:** 2020-09-30

**Authors:** Oswaldo Lorenzo-Quiles, João F. Soares-Quadros, Johanna E. Abril

**Affiliations:** 1 Department of Didactics of Musical, Plastic and Corporal Expression, Faculty of Education and Humanities of Melilla, University of Granada, Melilla, Spain; 2 Department of Music, Federal University of Maranhão, São Luís, Brazil; 3 School of Humanities, Department of Research, Universidad de Las Américas, Quito, Ecuador; Anadolu University, TURKEY

## Abstract

Music is considered a vital element in young people’s lives. It functions as an important means for expressing the emotions and feelings they experience in a daily basis. As such, this study explores the music preferences of high school Brazilian students (*N* = 940), 530 female (56.9%) and 410 male (43.1%) participants between 14 and 20 years old (*M* = 16.14 years old, *SD* = 1.22). The main instrument for data collection was the Questionnaire on Musical Style Preferences, which was adapted to the Brazilian context and encompassed 33 different music styles. A principal component analysis resulted in five dimensions representing different musical styles: (1) Intense, (2) Unique, (3) Sophisticated, (4) Contemporary, and (5) Mellow. The results of this study reinforced theory of the five-factor model of musical preference. Results also suggest that Mellow music was the most preferred while Sophisticated music was the least preferred among participants. Regarding gender, male participants showed a greater preference towards Contemporary, Intense, and Sophisticated music, whereas women generally preferred Mellow and Unique. Regarding age, participants under 20 years old showed a greater preference towards Mellow musical styles as compared to older participants. On the one hand, regression analyses showed that preferences towards Intense music decrease with age. On the other hand, gender was a better predictor for music preferences than age. Although the results of this study correspond to those of previous studies, more research studies are necessary to further explain musical preferences within the Brazilian context.

## Introduction

Music is present in the lives of most people and is acquired and transmitted in different ways [1]. Research has shown music’s influence on the social, cultural, educative, and even religious development of contemporary society [2], and as such, knowing people’s musical preferences is paramount in understanding the factors associated with music consumption.

Music preference can be defined as the favorable or unfavorable, permanent or discontinuous affinity towards different types and styles of music [3, 4]. Soares-Quadros Jr. and Lorenzo [5] contend that when a musical preference becomes more stable and long-term, it transforms into what we know as musical taste. However, Bonneville-Roussy et al. [6] found evidences suggesting that musical taste could also change throughout people’s lives.

Several studies have sought to find a robust and validated structure of music preferences. Among those studies, two models for music preference are prominent. Rentfrow and Gosling [7] introduced a model using a Short Test of Music Preference (STOMP) based on 14 broad musical styles. Data converged into a four-factor structure of music preference: (1) Reflective and Complex (blues, jazz, classic, and folk music), (2) Intense and Rebellious (rock alternative and heavy metal music), (3) Upbeat and Conventional (country, soundtracks, religious, and pop music), and (4) Energetic and Rhythmic (rap/hip-hop, soul/funk, and electronic/dance music). A number of authors [8–10] replicated this model and found similar results despite the cultural adaptations made on the questionnaire, especially considering the musical styles used to build the models.

Rentfrow, Goldberg, and Levitin [11] developed a second model, which is largely present in the current literature regarding music preferences. This model, called MUSIC, converged into a five-factor structure: (1) Mellow (e.g., pop and R&B), (2) Unpretentious (e.g., country and rock and roll), (3) Sophisticated (e.g., classical and jazz), (4) Intense (e.g., heavy metal and punk), and (5) Contemporary (e.g., rap and electronic). This model has been widely used in different studies, which have shown similar results around the world [12, 13]. In recent years, a revised version of the STOMP model was developed. The new STOMP-R model captures the dimensional structure of the MUSIC model through the inclusion of more musical styles [6, 14].

Other authors have challenged the number of dimensions of musical preferences and of musical styles used to build the models. In Germany, Schäfer and Sedlmeier [15] examined the musical preferences of 507 participants. Their analysis yielded six dimensions using 25 different music styles. In the United Kingdom, Colley [16] also found a six-dimensions structure, this time using only 14 musical styles. In Brazil, Herrera, Soares-Quadros Jr, and Lorenzo-Quiles [17] used 73 musical styles and identified nine musical dimensions. Lastly, North [18] conducted a study with people around the world and used 43 musical styles, which yielded a structure of 10 dimensions. Given the variations between studies, it is possible that the number of musical styles considered might directly influence how many musical dimensions are derived.

Why do people prefer one music style to another? This question has guided a number of recent studies [18–20] in which authors contended that certain elements can foster peoples’ inclination towards a determined musical style. For example, Jungaberle, Verres, and DuBois [21] argued that there is a generalized preference for music played at a comfortable volume level, in moderate time, and with a balance between simplicity and complexity. Other authors contended that music preferences are usually associated with: (a) familiarity with and repetitive hearing of music [22]; (b) social and cultural influences [23, 24]; (c) personality [9, 25, 26]; (d) music’s function and uses [27–29]; (e) the listener’s social class [5, 30]; and (f) the listener’s religious beliefs [19, 31, 32].

Additionally, other studies have examined musical preferences as they relate to age. Several studies have shown that musical preferences and their degree of intensity are directly related to age [14, 18]. For example, Way et al.’s study [33] indicates that adolescence is a crucial period for the development of musical identity. Levitin [34] highlighted that people remember the music from their adolescence because it corresponds to a time of self-discovery. Furthermore, it has a strong emotional influence because neurotransmitters “label” those experiences as something important.

On the other hand, Delsing, Ter Bogt, Engels, and Meeus [24] suggest that increasing stability of musical preferences throughout peoples’ lives might be associated with their personal stability and the formation of their individual and social identities. Thus, aspects such as marriage, family, and professional career, are factors that, associated with psychosocial development, can provoke a progressive departure from the uses given to music during adolescence [6]. Similarly, several authors have considered adolescence to be a crucial moment for the development of preferences that become musical tastes [22, 34]. As Bonneville-Roussy, Rentfrow, Xu, and Potter [6] argued, the meaning of music in the lives of people seems to increase in adolescence, gradually decreasing after that time. Accordingly, Levitin [34] pointed out that although there is no age limit for the development of new musical tastes, they often become strong and fixed between ages 18 and 20.

Bonneville-Roussy et al. [6] argued that a possible explanation for age differences in musical preferences might lie within the listeners’ personality differences. Related factors, such as neuroticism, are comparatively higher in adolescence than in adulthood. Additionally, the same authors noted that preference towards certain musical qualities (i.e., intensity, contemporary) decreases with age, while others tend to increase (i.e., unpretentious, sophisticated).

Studies on music preference have also considered gender as an analytical dimension [18, 35], but have not shown conclusive results of gender associated with music preferences. Nevertheless, there is some evidence which indicates that women have a preference for “softer” musical styles such as pop, whereas men prefer “heavier” styles such as rock [19]. Accordingly, Soares-Quadros Jr, Lorenzo-Quiles, Herrera, and Santos [36] conducted a study in Brazil with adolescents in which male participants showed greater preference towards “exciting music” (hip-hop and rap). In contrast, female participants showed greater preference towards popular styles of mass consumption (arrocha, brega, international, Brazilian popular music, and sertanejo styles) as well as gospel, a more conventional style.

Other authors focusing on gender-related musical preferences [13, 14, 18] contended that one of the main differences between men and women lies in how they use music. Accordingly, North and Hargreaves [22] argue that men, more than women, listen to one or another type of music because of preconceived societal opinions. This may be why men prefer heavy styles, such as rock, heavy metal, or rap, which they may view as aligned with traditional values of masculinity and virility. The authors also maintained that women, more than men, prefer music that satisfies emotional and physical needs. In this sense, Gouveia, Pimentel, Santana, Chaves, and Paraíba [8] have argued that women are more likely to adhere to normative and emotional values, whereas men tend to be liberal and in search of sensations. Similarly, North [18] has stated that men tend to use music to create an image of themselves and a sense of belonging within their social groups, whereas women use music for their own pleasure, to express feelings and emotions, and to alleviate loneliness.

Another line of research on gender and musical preference is related to attitudes towards music. Research has shown that women generally have a more positive attitude towards music than men [22]. However, Hui [37] noted that men are more likely to listen to music than women. On the other hand, Lorenzo-Quiles, Pérez-Garrido, and Soares-Quadros Jr [35] showed a relationship between environmental aspects and musical preferences regarding gender, except among professional music students.

The literature presented shows a variety of elements that strongly associated with people’s musical preferences peoples’ musical preferences, with age and gender seeming to be highly influential during adolescence. The purpose of this study was to examine the individual differences in age- and gender-related music preferences of Brazilian students. Considering the limited number of studies regarding music preferences within the Brazilian context, this study would allow us to compare the musical behavior of a large group within this population to similar studies conducted in different countries. Furthermore, some questions that guided this investigation were: How are students’ musical preferences structured? What are the main types of music they listen to? How can gender and age predict their musical preferences?

## Material and methods

### Participants

To carry out this study, approvals from the schools’ administration and higher administration entities were required. As most students were minors, a formal document requesting the consent to participate in this survey was sent to parents or guardians. Thus, students who returned signed consents composed the sample. Furthermore, the Ethics committee of the Secretary of Education of the State of Espírito Santo (Brazil) assessed this research before its application, acting as an institutional review committee for all aspects related to this research, including the instruments used with the participants. Thereby, this committee approved this study and guaranteed that it met the necessary ethical conditions.

The final sample was formed of 940 students (*N*_*female*_ = 530; *N*_*male*_ = 410; *χ*^*2*^ = 15.319, *p <* .001), ages ranged from 14 to 20 years old (*M* = 16.14, *SD* = 1.22; *χ*^*2*^ = 620.181, *p <* .001), from all public secondary schools (*N*_*School*_ = 13) in the city of Vitória (Brazil). Students were selected from the three high school grade levels in the Brazilian educational system: 35.5% first-year students; 32.6% second-year students; 31.9% third-year students (*χ*^*2*^ = 2.102, *p =* .35).

### Instrument

For this study, we implemented an adapted version of the Questionnaire on Musical Style Preferences [38]. A pilot study conducted with 89 students from high schools in Vitória informed the development of the adapted questionnaire and the selection of musical styles. Independent judges with expertise in the field examined a draft of the survey [39]. This group of 12 experts in music education, musicology, ethnomusicology, and general education from Brazilian universities, examined the research instrument to check for its suitability, congruence, and relevance. Additionally, a Cronbach’s alpha coefficient (*α* = .849) [40] was calculated to measure the internal consistency of the survey items.

The final version of the questionnaire was divided into two sections. Section 1 was used to collect demographic information about the participants (school, grade, gender, and age). Section 2 focused on how often the students listened to 33 musical styles present in the Brazilian and international musical culture. Using a Likert-type scale (1 = never; 5 = always), students indicated their listening habits and musical preferences. Additionally, students were given the option to check “I do not know this musical style. I can’t answer it,” if they did not have enough information to answer the question”.

### Procedure

The questionnaire was administered in-person after receiving approval from the schools’ administration and higher administration entities. The researchers oversaw the administration of the questionnaire in order to clarify any doubts participants had about the questions and content. Each session lasted approximately 50 minutes.

### Analysis

Data collected were entered and analyzed into SPSS 24.0. The independent variables chosen for this study were gender and age. Levene’s test for equality showed homogeneous variances (*p* > .05) when considering both variables. Based on this, and when the size of the sample was sufficiently large, we performed Pearson’s correlations and Analysis of Variance tests with Bonferroni adjustments for multiple comparisons. Additionally, partial eta-squared (*η*_*p*_^*2*^) values were calculated to determine the magnitude of the differences between groups, regarding gender and age variables. Finally, a hierarchical regression analysis was performed to determine whether gender and age could predict musical preferences.

## Results

### Structure of musical preferences

**Fig 1:**
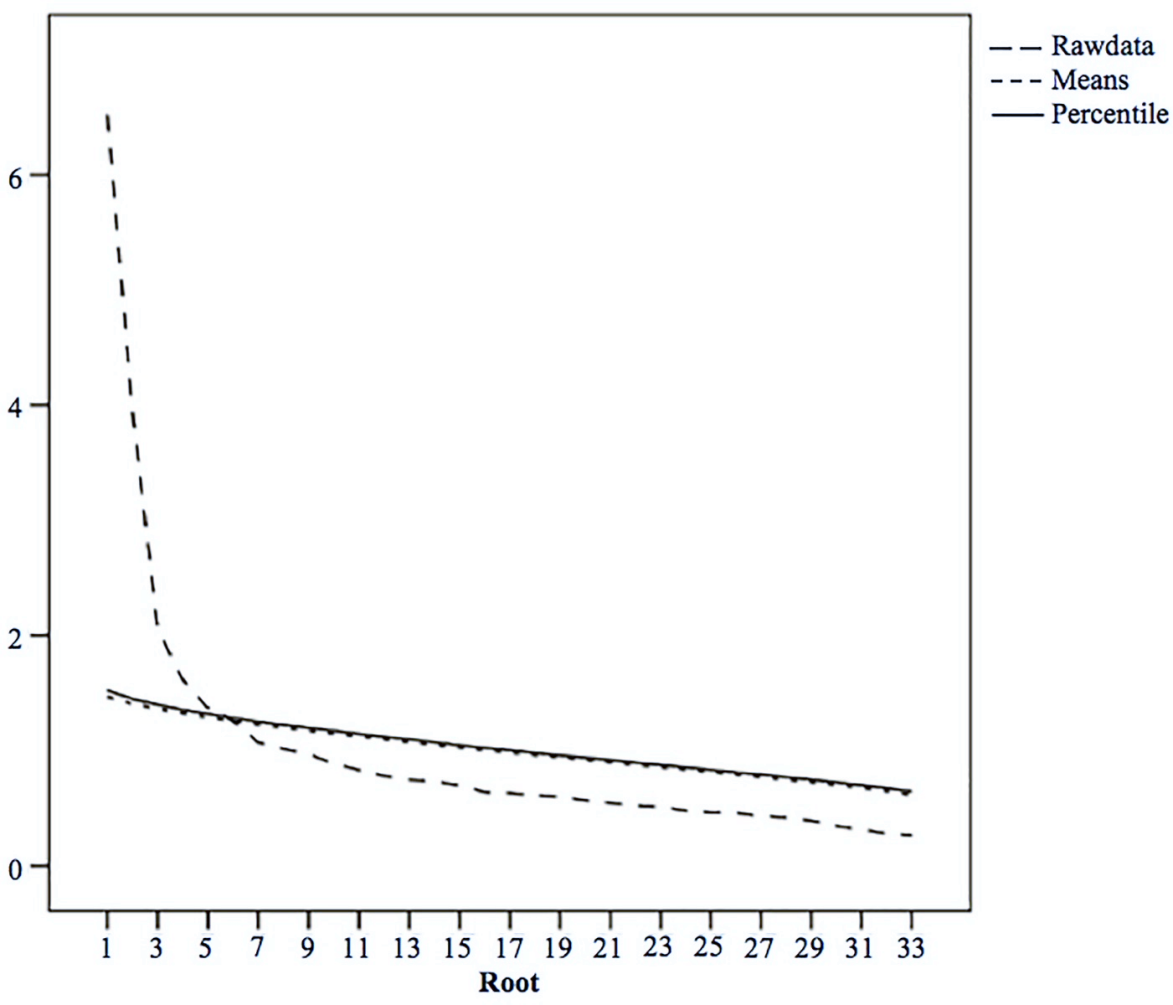
Parallel analysis scree-plot for the 33 music styles evaluated.

A Principal Component Analysis (PCA) with Varimax Rotation and Kaiser Normalization was conducted to analyze the underlying structure of the scale. Both the Kaiser-Meyer-Olkin sample adequacy measure (*KMO* = .870) and the Barlett sphericity test (*χ*^*2*^ *=* 6171.443, *p* < .001) suggested that the sample was factorable [41]. The retained factors were extracted through parallel analysis (Monte Carlo simulation) and scree-plot analysis [42] (Fig 1). This analysis yielded five dimensions explaining 46.96% of the total variance (Table 1).

**Table 1:** Factor loadings based on a principal components analysis with Varimax rotation for 33 music styles in a frequency of listening scale.

Music style	1	2	3	4	5
**Metal**	.77	-.04	.23	.11	.01
**Rock**	.76	-.11	.03	.11	.19
**Gothic**	.70	.00	.18	-.07	-.03
**Core**	.68	.01	.14	-.06	.07
**Punk**	.63	-.07	.14	.16	.07
**Pop-rock**	.58	-.02	.09	.22	.52
**Electronic**	.41	.29	.01	.33	.34
**Axé-music**	-.03	.73	-.03	.15	.02
**Sertanejo**	.01	.68	-.07	.06	.30
**Forró**	-.06	.67	.15	.00	.16
**Pagode**	-.25	.62	-.09	.34	.06
**Funk**	-.31	.59	-.08	.40	-.16
**Samba**	-.14	.57	.17	.23	.15
**Country**	.22	.46	.34	-.10	.20
**Tecno-brega**	.19	.40	.14	.05	-.14
**Brega**	.19	.35	.34	-.12	-.12
**Folkloric**	-.11	.33	.28	-.16	.25
**Chorinho**	.02	.10	.75	.12	.04
**Bossa-nova**	.16	.03	.67	.13	.11
**Jazz**	.20	.04	.60	.20	.21
**Classic**	.13	-.01	.56	.00	.31
**Blues**	.29	-.10	.56	.03	-.04
**Frevo**	-.03	.17	.51	.00	.10
**Surf music**	.27	.11	.39	.27	-.06
**Brazilian popular music**	.22	.12	.35	.25	.34
**Hip-hop**	.00	.12	.00	.72	.17
**Rap**	.07	.09	.15	.69	.03
**Reggae**	.24	.29	.23	.47	-.09
**Stronda music**	.05	.09	.25	.37	.05
**Pop**	.33	.04	.05	.36	.64
**Romantic**	.02	.29	.16	-.09	.62
**Soundtracks**	.22	.02	.37	.13	.55
**Gospel**	-.25	.11	.14	-.32	.38
**% of Variance**	19.76	11.94	6.23	4.89	4.14
**Reliability (*α*)**	.82	.78	.74	.62	.53

Dimension 1, identified as Intense, comprised seven musical styles (metal, rock, gothic, punk, pop-rock, and electronic music) characterized by their loud, forceful, and energetic nature. Dimension 2, identified as Unique, consisted of unique, singular, specific musical styles from Brazilian culture (axé, sertanejo, forró, pagode, funk, samba, country, tecno-brega, brega, and folkloric music) often linked to dancing and described as light music, songs of low musical complexity and massive character. Dimension 3, Sophisticated, included eight musical styles (chorinho, bossa-nova, jazz, classic, blues, frevo, surf music, and Brazilian popular music) perceived as complex, intelligent, and inspiring. Dimension 4, Contemporary, consisted of four musical styles (rap, hip-hop, reggae, and stronda music) considered to be rhythmic, percussive, and usually linked to issues of social justice and inequalities among lower SES populations. Finally, dimension 5, identified as Mellow, comprised four musical styles (pop, romantic, soundtracks, and gospel music) perceived as emotional, smooth, and relaxing in nature.

Pearson correlations showed significant interactions between all dimensions, except for Intense and Unique. Table 2 shows moderate positive correlations between Intense and Sophisticated, Unique and Contemporary, Sophisticated and Contemporary, and Sophisticated and Mellow [43].

**Table 2:** Pearson correlations for musical dimensions.

	Intense	Unique	Sophisticated	Contemporary	Mellow
**Intense**	1				
**Unique**	.024	1			
**Sophisticated**	.465***	.239***	1		
**Contemporary**	.310***	.412***	.402***	1	
**Mellow**	.351***	.274***	.415***	.222***	1

***p < .001

#### General results on music preference

**Fig 2:**
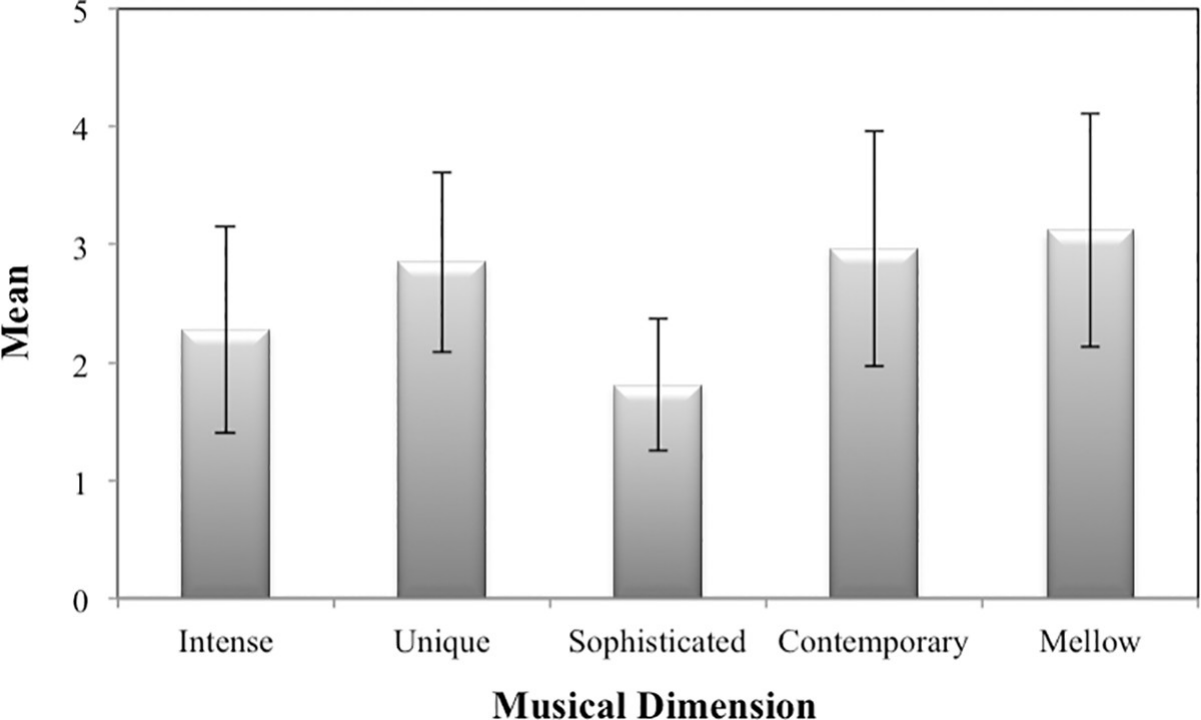
Means and standard deviations regarding the listening frequency to the five musical dimensions.

Participants were more likely to listen to Mellow music (*M* = 3.12, *SD* = .99), followed by Contemporary music (*M* = 2.97, *SD =* 1.00). On the other hand, Sophisticated music (*M* = 1.81, *SD* = .56) seemed to be the least popular music style (Fig 2).

#### Gender and age

**Fig 3:**
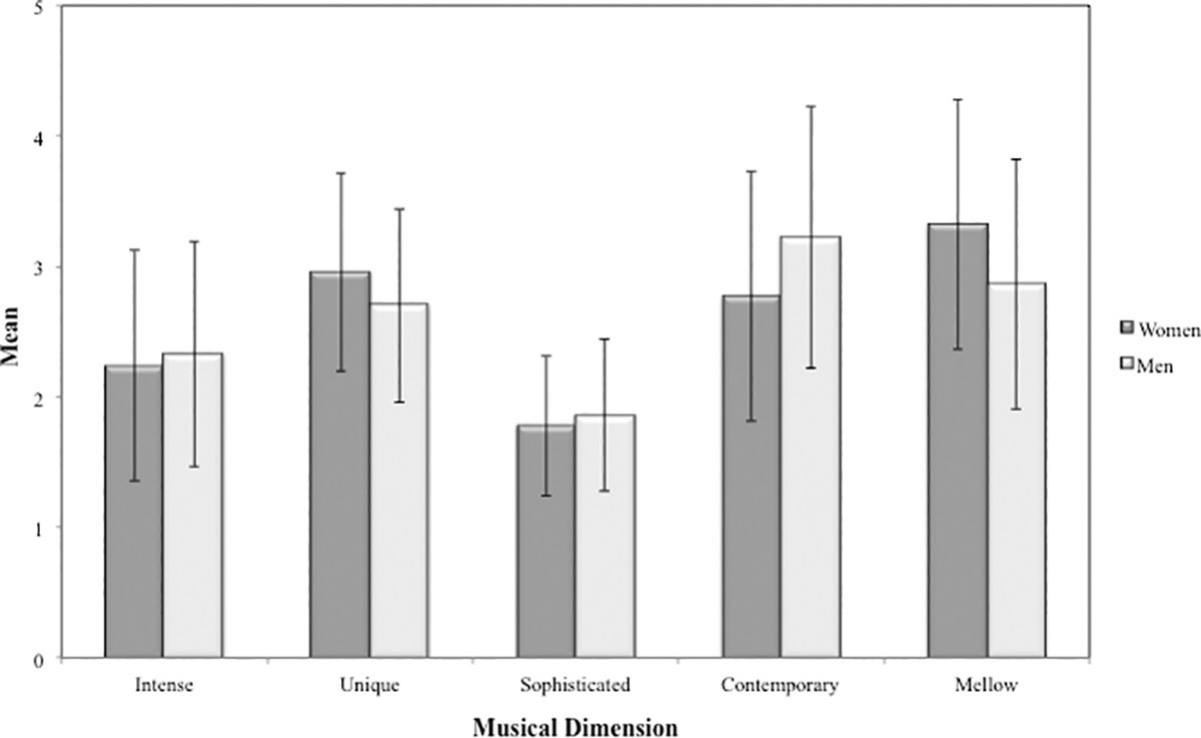
Descriptive statistics for musical preferences regarding gender.

Descriptive analyzes regarding gender (Fig 3) showed a higher listening frequency of Contemporary musical styles among male students (*M =* 3.09, *SD =* .078), whereas female students listened to Mellow musical styles more frequently (*M =* 3.04, *SD =* .082). Both, male and female participants seemed to overlook Sophisticated music styles (*M*_*Male*_ *=* 1.84, *SD*_*Male*_ *=* .045; *M*_*Female*_ *=* 1.65, *SD*_*Female*_ *=* .048).

Concerning age (Table 3), all groups showed greater preference towards Mellow music styles, except for 18 and 20-year-old participants, who showed greater preference towards Contemporary and Unique music styles, respectively. Sophisticated music was the least listened to by all groups.

**Table 3:** Descriptive statistics for musical preferences regarding age.

Dimensions	14		15		16		17		18		19		20	
	*M*	*SD*	*M*	*SD*	*M*	*SD*	*M*	*SD*	*M*	*SD*	*M*	*SD*	*M*	*SD*
**Intense**	2.32	.10	2.39	.06	2.31	.05	2.25	.06	2.14	.11	2.09	.19	1.68	.25
**Unique**	2.71	.09	2.77	.05	2.89	.04	2.90	.05	2.89	.10	2.51	.16	2.55	.22
**Sophisticated**	1.76	.07	1.84	.04	1.83	.03	1.83	.04	1.87	.07	1.72	.12	1.34	.16
**Contemporary**	2.98	.11	3.05	.07	2.98	.06	3.03	.06	3.11	.12	2.53	.21	2.31	.28
**Mellow**	2.95	.11	3.14	.07	3.11	.06	3.15	.06	2.95	.12	2.82	.21	2.22	.27

Analysis of variance showed significant differences and varied effect sizes between musical preferences for both gender and age, as well as for the interaction between both variables (Table 4). Comparisons between gender groups revealed significant differences and varied effect sizes for Contemporary (*t* = .464, *p <* .001, *η*_*p*_^*2*^ = .017), Intense (*t* = .237, *p =* .022, *η*_*p*_^*2*^ = .006), and Sophisticated (*t* = .184, *p =* .005, *η*_*p*_^*2*^ = .008), which were more preferred among male students. On the other hand, Unique (*t* = .217, *p =* .015, *η*_*p*_^*2*^ = .006) and Mellow (*t* = .262, *p =* .020, *η*_*p*_^*2*^ = .006) dimensions showed significant differences with small effect sizes, and were more preferred among female students. Regarding age, group comparisons showed significant differences and small effect sizes for Unique, Contemporary, and Mellow dimensions. However, multiple comparisons with Bonferroni adjustments indicated significant differences only for Mellow music styles, which were more popular among 15-year-old [*t*_(6,933)_ = .922, *p =* .020], 16-year-old [*t*_(6,933)_ = .896, *p =* .026], and 17-year-old [*t*_(6,933)_ = .932, *p =* .017] participants than the 20-year-old group.

**Table 4:** ANOVA and effect sizes (*η_p_^2^*) for musical styles by gender and age.

Dimension	Gender			Age			Gender x Age		
	*F*	*p*	*η*_*p*_^*2*^	*F*	*p*	*η*_*p*_^*2*^	*F*	*p*	*η*_*p*_^*2*^
**Intense**	5.299	.022	.006	2.083	.053	.013	.939	.466	.006
**Unique**	5.961	.015	.006	2.137	.047	.014	.822	.553	.005
**Sophisticated**	7.878	.005	.008	1.918	.075	.012	2.315	.032	.015
**Contemporary**	16.338	< .001	.017	2.168	.044	.014	1.053	.390	.007
**Mellow**	5.456	.020	.006	2.768	.011	.018	2.532	.019	.016

**Fig 4:**
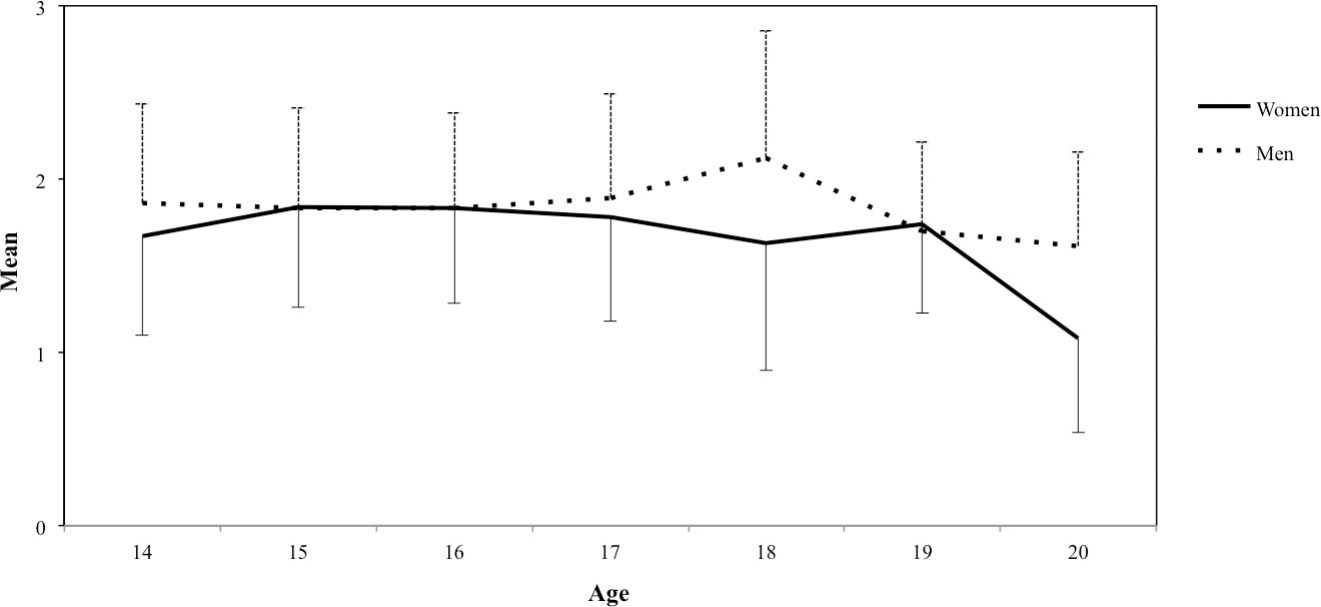
Interaction between gender and age variables for Sophisticated styles.

Analyses of the interaction between the independent variables showed significant differences and small effect sizes for Sophisticated and Mellow music styles. In addition, post-hoc comparisons showed that 18-year-old male participants had a stronger preference toward Sophisticated styles (*t* = .487, *p <* .001) than their female counterparts (Fig 4).

**Fig 5:**
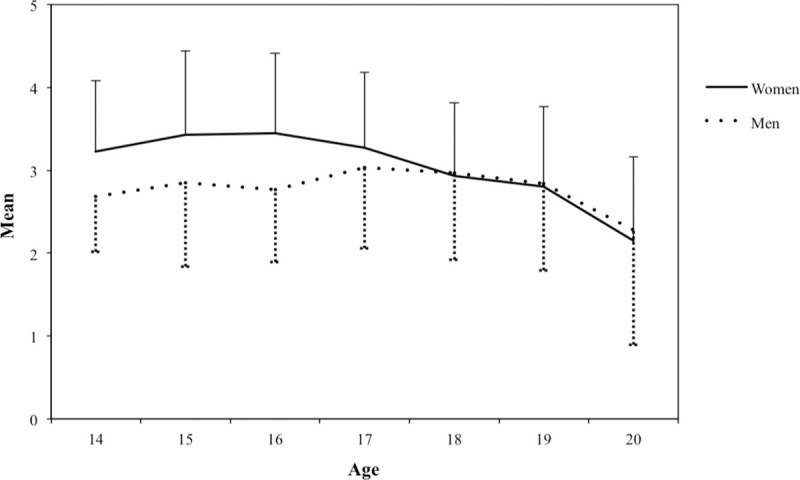
Interaction between gender and age variables for Mellow styles.

On the other hand, women ages 14 (*t* = .548, *p =* .014), 15 (*t* = .580, *p <* .001), 16 (*t* = .681, *p <* .001), and 17 (*t* = .241, *p =* .044) had a greater preference towards Mellow styles than their male counterparts (Fig 5). Furthermore, it was found that Mellow styles were more popular among 16-year-old female participants (*t* = .525, *p =* .040) than among 18-year-old women.

Several stepwise linear regressions were performed to verify how gender and age could predict the participants’ musical preferences. The regression model considered age at step 1 and gender at step 2, which accounted for 15.32% of the variance. As can be seen at Table 5, age was significant only for the Intense dimension (*t =* -3.051, *p* = .002), which suggests that the musical preference towards this type of music decreases over time. When the gender variable was included in the model, the amount of the explained variance increased significantly towards Unique (*t = -*5.176, *p* < .001), Sophisticated (*t =* 2.294, *p* < .022), Contemporary (*t =* 7.059, *p* < .001), and Mellow music (*t = -*7.134, *p* < .001).

**Table 5:** Step-wise linear regressions of musical dimensions with age and gender.

Musical Dimension	Step 1. Age		Step 2. Gender (1 = female)		*ΔF*
	*R*^*2*^	*β*	*R*^*2*^	*β*	
**Intense**	.009	-.099	.003	.054	2.802
**Unique**	.000	.026	.028	-.167	26.791***
**Sophisticated**	.001	-.23	.005	.075	5.260*
**Contemporary**	.002	-.049	.050	.225	49.835***
**Mellow**	.002	-.052	.053	-.227	50.892***

*p < .05

***p < .001.

### Discussion and conclusions

The purpose of this study was to analyze the music preferences of a group of Brazilian high school students. A PCA was performed in order to determine the underlying structure of 33 musical styles. We identified five dimensions (Intense, Unique, Sophisticated, Contemporary, and Mellow) that corroborated with the MUSIC model by Rentfrow et al. [11].

The first dimension, Intense, characterized by loud, forceful, and energetic music, was consistent with previous studies [15, 44, 45]. The second dimension, Unique, comprised uncomplicated, unaggressive, soft music, consistent with the findings of different authors [14, 46]. Additionally, it is worth noticing that most of the music styles comprising this dimension are originally from Brazil and connected to dance. These are similar to Música de Massa dimension by Gouveia et al. [8], and Brazilian Mainstream Music dimension by Herrera et al. [17]. The third dimension, Sophisticated, consisted of music styles perceived as complex, intelligent, and inspiring, which related to findings from other studies [9, 16, 47]. The fourth dimension, Contemporary, comprised rhythmic and percussive styles connected to issues of social justice and opportunities for lower SES populations, which converged with the findings of several authors [6, 15, 16, 32]. Lastly, the fifth dimension, Mellow, comprised relaxing musical styles with smooth rhythms and sentimental vocals, consistent with the dimensions Upbeat and Conventional found by prior studies [7, 10, 48], as well as with dimension Upbeat found by Herrera et al. [17]. Despite the variability in dimension composition, we argue that the results of this study confirm the five-factor structure of musical preferences proposed by Rentfrow et al. [11]. Nevertheless, further studies are needed to support this model.

Surprisingly, data showed that Mellow styles (gospel, pop, romantic, and soundtracks) were more likely to be chosen among participants. This corroborated findings from Clark and Giacomoantonio [48], but it contrasted findings from some authors [14, 16, 45, 49], who contended that rock styles are the most popular among adolescents. Aside from the musical features of Mellow styles, it is possible that their popularity among participants responded to two elements motivating the participants’ choices. First, the participants’ familiarity with gospel music could be due to the strong presence of protestant religions within the context. According to data from Brazilian government [50], the State of Espírito Santo (of which Vitória is the capital) has the highest number of Protestants, having doubled between 2000 and 2010 [51]. Second, the strong preference for pop music among adolescents aligns with the findings of two previous studies [15, 52].

On the other hand, the overall rejection of Sophisticated styles might be related to a lower tolerance, curiosity, and openness to a wide range of musical styles, which Hargreaves [53] defines as open-earedness. These results support the hypothesis that open-earedness declines during adolescence and is restructured in adulthood [36]. Another explanation for these results could be the unfamiliarity of the participants with Sophisticated music styles [54]. Different studies have observed that the listening to unfamiliar music decreases significantly with age [55, 56], which might show that Brazilian adolescents know little about styles such as blues, classical music, and jazz. Moreover, styles such as bossa-nova and chorinho, despite being Brazilian, are more popular among adults and have not been widely disseminated within adolescents and young adults. These results show the need to create public cultural policies that insert adolescents and young adults in cultural scenes that allow them to experience these types of music.

When studying the differences between gender groups, male participants showed a stronger preference for Contemporary, Intense, and Sophisticated music, whereas female participants showed more preference towards Mellow and Unique. These results corroborate studies from different countries, which show that men usually prefer sophisticated musical styles with intense rhythms and linked to behaviors that strengthen ties within peer groups and challenge imposed social rules [8, 17, 18, 22], whereas women usually prefer music that emphasizes positive emotions as well as dancing activities [6, 9, 10, 16].

Regarding age, the results are not conclusive, which may be due to the specific characteristics of Brazilian youth culture. The lower preference for Mellow styles among 20-year-old participants as compared to younger groups may be related to what Levitin [34] defines as crystallization of musical preferences. He considers the period between 18 and 20 years as a limited period of time for the acquisition of new musical preferences, in which preferences are transformed into musical taste. These tastes establish a long-lasting preference [17]. Delsing et al. [24] contend that the increase in the stability of preferences may be associated with personal maturity and the formation of individual identity. Thus, musical styles that undergo frequent stylistic and that are strongly linked to music industry trends, tend to be preferred among young individuals.

Analyses of the interaction between variables presented results worth noting for two out of the five dimensions present in the model. First, younger girls showed a strong preference for Mellow styles when compared to boys of the same age as well as older girls. This difference may be attributed to adolescence being a period of self-discovery in which the individual undergoes great changes at the physical, mental and social level [57]. Important memories are developed during adolescence. This process is particularly prevalent around 16 years old, considered the apex of reminiscence [58]. Although the results of this study align with previous research [11, 12, 34], who claim that adolescents generally prefer music associated with dreamy, romantic, sad, and inspiring emotional states, further research is needed to support these findings.

Second, 18-year-old male and female participants showed different behaviors for the Sophisticated dimension, which was highly preferred among men. Although this is partially consistent with the statement by Bonneville-Roussy et al. [6], who suggest that the preference for this type of music increases considerably during adolescence, results of the present study lack enough evidence to support such statement. Hence, further studies are needed to clarify this phenomenon.

Finally, the regression analysis showed gender as a stronger predictor for the participants’ musical preferences than age. The results obtained reinforce the hypothesis of a greater preference of women towards light and popular music, while men preferred sophisticated and rhythmic musical styles, as suggesting in prior studies [16, 25]. However, this analysis indicated a possible association of the age variable with preferences of men towards hard and intense music (e.g., meal, rock, and punk). According to Bonneville-Roussy et al. [6], the preference of this type of music tends to reduce over time, independently of the gender. Frequently, hard and intense music is defined as a significant thing in the lives of adolescents, because it helps them to strengthen their feelings of social disagreement, to form their identity, and to establish their ways of interpersonal relationship [15, 24, 59]. With the advancement of age towards a more mature stage, the feelings and the self-concepts become more stables due to important changes in their life regarding the relevance of their role inside of society, such as entering the labor market, forming a family or preparing to start an academic career [60, 61]. Thus, it is possible to believe that adults use music more to emotional regulation or stimulation than as a way of identity development [6]. However, given that the *R*^*2*^ values obtained in this study were small, it is necessary to take the results presented here with caution [13].

### Limitations and suggestions for future research

This study has provided valuable information to develop a better understanding of musical preferences among Brazilian adolescents. However, the lack of studies concerning the Brazilian context has limited our ability to make adequate comparisons between the findings of the study and the musical styles specific to the Brazilian culture. Additionally, it is necessary to explain further how the dimensions present in the literature reviewed for this study have been created and justified in regard to their stylistic attributes.

Another limitation for this study was our inability to assess participants’ musical preferences through an excerpt-based questionnaire. Although originally proposed in the project, the administrators at the participant sites rejected this methodology because it was thought that the use of music could disturb teachers in other classes. Therefore, it is necessary to conduct studies that allow the collection of excerpt-based data since it would minimize biases related to individual perceptions of different musical styles among participants.

Regarding the dimensions found in this study, Contemporary and Mellow musical styles showed internal consistency indexes considered to be questionable and poor, respectively [62]. This could be due to the different levels of popularity among the musical styles encompassed in each dimension and consequently, the high variability in the listening frequency of those styles. Another influencing factor could be the limited number of styles within each dimension. Therefore, it is possible that the inclusion of more musical styles, equally popular and with similar characteristics to those chosen for the dimensions, would improve the internal consistency of the Contemporary and Mellow musical dimensions.

For this study, we chose gender and age as predictive variables. According to Anderson et al. [63], musical preferences could be better analyzed when they are combined with measures of habitual listening behaviors, which would predict the listeners’ personalities. Thus, future studies should include variables such as personality and music consumption habits in addition to those used in the present study.

Lastly, a larger sample size encompassing different age groups would provide a more generalized understanding of musical preferences and therefore, musical behaviors among Brazilian people. Second, it is necessary to conduct longitudinal studies in order to understand how music preferences change throughout different points in time. Lastly, it would be beneficial to evaluate the music preferences of people through alternative methodologies beyond self-reported assessments, namely excerpt-based assessments.

## References

[1] North A, Krause A, Sheridan L, Ritchie D. Energy, typicality, and music sales: a computerized analysis of 143,353 pieces. Empir Stud Arts. 2017 1 22; 35(2): 214–229.

[2] Solli H, Rolvsjord R, Borg M. Toward understanding music therapy as a recovery-oriented practice within mental health care: a meta-synthesis of services users’ experiences. J Music Ther. 2013 12 1; 50(4): 244–73. 10.1093/jmt/50.4.244 25014667

[3] Hargreaves D, North A, Tarrant M. How and why do musical preferences change in childhood and adolescence? In: McPherson G, editor. The child as musician: a handbook of musical development. Oxford, UK: Oxford University Press; 2015 p. 303–22.

[4] Pimentel C, Gouveia V, Coelho L Jr, Athayde R, Lima T. [Music preference and sensation seeking among youngsters]. Psicol. 2014; 34(1): 4–17. Brazilian.

[5] Soares-Quadros JF Jr, Lorenzo-Quiles O. Preferência musical: uma revisão dos fatores extramusicais que influenciam na escolha de músicas por ouvintes In: Molinari P, editor. Música, educação e cultura: tecituras e tessituras no Nordeste Brasileiro. São Paulo: Editora Faccapm; 2016 p. 63–87. Brazilian.

[6] Bonneville-Roussy A, Rentfrow P, Xu M, Potter J. Music through the ages: Trends in musical engagement and preferences from adolescence through middle adulthood. J Pers Soc Psychol. 2013 7 29; 105(4): 703–17. 10.1037/a0033770 23895269

[7] Rentfrow P, Gosling S. The do re mi’s of everyday life: The structure and personality correlates of music preferences. J Pers Soc Psychol. 2003; 84(6): 1236–56. 10.1037/0022-3514.84.6.1236 12793587

[8] Gouveia V, Pimentel C, Santana N, Chaves W, Paraíba C. [Short test of music preference (STOMP): evidences of its factor validity and reliability]. Psico. 2008 8 29; 39(2): 201–10. Brazilian.

[9] Langmeyer A, Guglhör-Rudan A, Tamai C. What do music preferences reveal about personality? A cross-cultural replication using self-ratings and ratings of music sample. J Individ Differ. 2012; 33(2): 119–30.

[10] Zweigenhaft R. A do re mi encore: A closer look at the personality correlates of music preferences. J Individ Differ. 2008; 29(1): 45–55.

[11] Rentfrow P, Goldberg L, Levitin D. The structure of musical preferences: A five-factor model. J Pers Soc Psychol. 2011; 100(6): 1139–57. 10.1037/a0022406 21299309 PMC3138530

[12] Rentfrow P, Goldberg L, Stillwell D, Kosinski M, Gosling S, Levitin D. The song remains the same: A replication and extension of the MUSIC model. Music Percept. 2012; 30(2): 161–85. 10.1525/mp.2012.30.2.161 24825945 PMC4016970

[13] Schäfer T, Mehlhorn C. Can personality traits predict musical style preferences? A meta-analysis. Pers Individ Differ. 2017 10 1; 116: 265–73.

[14] Fricke K, Herzberg P. Personality and self-reported preference for music genres and attributes in a German-speaking sample. J Res Pers. 2017; 68: 114–23.

[15] Schäfer T, Sedlmeier P. From the functions of music to music preference. Psychol Music. 2009 3 10; 37(3): 279–300.

[16] Colley A. Young people’s musical taste: relationship with gender and gender-related traits. J Appl Soc Psychol. 2008 7 24; 38(8): 2039–55.

[17] Herrera L, Soares-Quadros JF Jr, Lorenzo-Quiles O. Music preferences and personality in Brazilians. Front Psychol. 2018 8 21; 9: 1488 10.3389/fpsyg.2018.01488 30186197 PMC6113570

[18] North A. Individual differences in musical taste. Am J Psychol. 2010; 123(2): 199–208. 10.5406/amerjpsyc.123.2.0199 . 20518436

[19] Ok Ü, Erdal B. Religious and demographic indicators of music preference in a Turkish sample. Music Sci. 2015 11 12; 19(1): 23–43.

[20] Thomas K. Music preferences and the adolescent brain: a review of literature. Update Appl Res Music Educ. 2016; 35(1): 47–53. Epub 2015 Apr 20.

[21] Jungaberle H, Verres R, DuBois F. New steps in musical meaning the metaphoric process as an organizing principle. Nord J Music Ther. 2001; 10(1): 4–16.

[22] North A, Hargreaves D. The social and applied psychology of music. New York: Oxford University Press; 2008.

[23] Brittin R. Young listeners’ music style preferences: Patterns related to cultural identification and language use. J Res Music Educ. 2014; 61(4): 415–30. Epub 2013 Nov.

[24] Delsing M, Ter Bogt T, Engels R, Meeus W. Adolescents’ music preferences and personality characteristics. Eur J Pers. 2008; 22(2): 109–30. Epub 2007 Nov 07.

[25] Dobrota S, Ercegovac I. The relationship between music preferences of different mode and tempo and personality traits–implications for music pedagogy. Music Educ Res. 2015; 17(2): 234–47. Epub 2014 Jul 07.

[26] Greenberg DM, Matz SC, Schwartz HA, Fricke KR. The self-congruity effect of music. J Pers Soc Psychol. 2020 10.1037/pspp0000293 . Epub 2020 Jul 2. 32614219

[27] Getz L, Chamorro-Premuzic T, Roy M, Devroop K. The relationship between affect, uses of music, and music preferences in a sample of South African adolescents. Psychol Music. 2012; 40(2): 164–78. Epub 2011 Jan 14.

[28] Schäfer T, Tipandjan A, Sedlmeier P. The functions of music and their relationship to music preference in India and Germany. Int J Psychol. 2012 6 21; 47(5): 370–80. 10.1080/00207594.2012.688133 22721000

[29] Shepherd D, Sigg N. Music preference, social identity, and self-esteem. Music Percept. 2015 6 01; 32(5): 507–14.

[30] Bourdieu P. Distinction: a social critique of the judgment of taste. London: Routledge; 2010.

[31] Boer D, Fischer R. Towards a holistic model of functions of music listening across cultures: A culturally decentered qualitative approach. Psychol Music. 2012; 40(2): 179–200. Epub 2011 Jan 14.

[32] George D, Stickle K, Rachid F, Wopnford A. The association between types of music enjoyed and cognitive, behavioral, and personality factors of those who listen. Psychomusicology. 2007; 19(2): 32–56.

[33] Way SF, Gil S, Anderson I, Clauset A. Environmental Changes and the Dynamics of Musical Identity. International AAAI Conference on Web and Social Media [Internet]. 2019 Jul 06 [cited 2020 Aug 27]; 13(1): 527–36. Available from: https://www.aaai.org/ojs/index.php/ICWSM/article/view/3250

[34] Levitin D. This Is Your Brain on Music: The Science of a Human Obsession. London: Atlantic Books; 2011.

[35] Lorenzo-Quiles O, Pérez-Garrido A, Soares-Quadros JF Jr. [Study about the preferences of musical style of Spanish students of higher education at the Conservatory of Music]. Musica Hodie. 2014 12 01; 14(1): 211–22. Brazilian.

[36] Soares-Quadros JF Jr, Lorenzo-Quiles O, Herrera L, Santos N. Gender and religion as factors of individual differences in musical preference. Music Sci. 2019; 23(4): 525–39. Epub 2018 May 14.

[37] Hui W. Music listening preferences of Macau students. Music Educ Res. 2009 11 03; 11(4): 485–500.

[38] Cremades R, Lorenzo-Quiles O, Herrera L. Musical tastes of Secondary School Student´s with different cultural backgrounds: A study in the Spanish North African city of Melilla. Music Sci. 2010 3 01; 14(1): 121–41.

[39] Escobar-Pérez J, Cuervo-Martínez A. Validez de contenido y juicio de expertos: una aproximación a su utilización. Av Medicion. 2008; 6(1): 27–36.

[40] Cronbach L. My current thoughts on coefficient alpha and successor procedures. Educ Psychol Meas. 2004 6 01; 64(3): 391–418.

[41] Dancey C, Reidy J. Statistics without maths for Psychology: using SPSS for Windows. London: Prentice Hall; 2004.

[42] Hayton J, Allen D, Scarpello V. Factor retention decisions in exploratory factor analysis: a tutorial on Parallel Analysis. Organ Res Methods. 2004 4 01; 7(2): 191–205.

[43] Schober P, Boer C, Schwarte LA. Correlation Coefficients: appropriate use and interpretation. Anaesth Analg. 2018; 126(5): 1763–68. 10.1213/ANE.0000000000002864 . 29481436

[44] Bonneville-Roussy A, Rust J. Age trends in musical preferences in adulthood: 2. sources of social influences as determinants of preferences. Music Sci. 2017 5 10; 22(2): 175–95.

[45] Vella E, Mills G. Personality uses of music, and music preference: The influence of openness to experience and extraversion. Psychol Music. 2016 8 01; 45(3): 1–17.

[46] Mellander C, Florida R, Rentfrow P, Potter J. The geography of music preferences. J Cult Econ. 2018 3 07; 42: 593–618.

[47] Knox D, MacDonald R. Broadcasting personalities: the relationship between occupation and music preferences in the BBC Radio programme Desert Island Discs. Psychol Music. 2017; 45(5): 645–64. Epub 2016 Oct 07.

[48] Clark S, Giacomantonio S. Music preferences and empathy: Toward predicting prosocial behavior. Psychomusicology. 2013; 23(3): 177–86.

[49] Dunn P, Ruyter B, Bouwhuis D. Toward a better understanding of the relation between music preference, listening behavior, and personality. Psychol Music. 2012; 40(4): 411–28. Epub 2011 Mar 16.

[50] Brazilian Institute of Geography and Statistics. Censo: Amostra–Religião [Internet]. 2010 [cited 2020 May 16]. Available from: https://cidades.ibge.gov.br/brasil/es/vitoria/pesquisa/23/22107?localidade1=0&indicador=22426&ano=2010&localidade2=32

[51] Araújo R. Proporção de evangélicos no Espírito Santo é maior do que no restante do país [Internet]. Jornal A Gazeta. 2012 6 29 [cited 2020 May 16]. Available from: http://gazetaonline.globo.com/_conteudo/2012/06/a_gazeta/minuto_a_minuto/1292361-proporcao-de-evangelicos-no-espirito-santo-e-maior-do-que-no-restante-do-pais.html

[52] Hargreaves D, Comber C, Colley A. Effects of age, gender, and training on musical preferences of British secondary school students. J Res Music Educ. 1995 10 01; 43(3): 242–50.

[53] Hargreaves D. The development of aesthetic reactions to music. Psychol Music. 1982; Special Issue: 51–4.

[54] Leblanc A. An interactive theory of music preference. J Music Ther. 1982 3 01; 19(1): 28–45.

[55] Kopiez R, Lehmann M. The ‘open-earedness’ hypothesis and the development of age-related aesthetic reactions to music in elementary school children. Br J Music Educ. 2008 6 11; 25(2): 121–38.

[56] Louven C. Hargreaves ‘open-earedness’: A critical discussion and new approach on the concept of musical tolerance and curiosity. Music Sci. 2016 2 18; 20(2): 235–47.

[57] Oerter R, Dreher E. Jugendalte In: Oerter R, Montada L, editors. Entwicklungspsychologie. Weinheim: Beltz; 2002 p. 258–318. German.

[58] Huron D. Sweet anticipation: music and the psychology of expectation. Cambridge, MA: MIT Press; 2006.

[59] Hällsten M, Edling C, Rydgren J. School’s out forever? Heavy metal preferences and higher education. Plos one. 2019 3 19; 14: e0213716. 10.1371/journal.pone.0213716 PMC6424403 30889202

[60] Harter S. The development of self-representations during childhood and adolescence In: Leary M, Tangney J, editors. Handbook of self and identity. New York: Guilford Press; 2003 p. 610–42.

[61] Steinberg L, Monahan KC. Age differences in resistance to peer influence. Dev Psychol. 2007; 43(6): 1531–43. 10.1037/0012-1649.43.6.1531 18020830 PMC2779518

[62] George D, Mallery P. SPSS for Windows step by step: A simple guide and reference. Boston; Allyn & Bacon; 2003.

[63] Anderson I, Gil S, Gibson C, Wolf S, Shapiro W, Semerci O, Greenberg DM. “Just the Way You Are”: Linking Music Listening on Spotify and Personality. Soc Psychol Personal Sci. 2020 Epub 2020 jul 10. 10.1177/1948550619865057 32577160 PMC7310997

