# Correction: Musical preferences of Brazilian high school students

**DOI:** 10.1371/journal.pone.0253353

**Published:** 2021-06-10

**Authors:** Oswaldo Lorenzo-Quiles, João F. Soares-Quadros, Johanna E. Abril

An additional affiliation is missing for the second author. João F. Soares-Quadros Jr. is also affiliated with Department of Music, Federal University of Ouro Preto, Ouro Preto, Minas Gerais, Brazil.

The ORCID iD is also missing for the second author. João F. Soares-Quadros Jr’s ORCID iD is: 0000-0003-3763-929X (https://orcid.org/0000-0003-3763-929X).
